# Early impact of RSV prevention on infant hospitalisations in Denmark

**DOI:** 10.1016/j.lanepe.2026.101750

**Published:** 2026-06-22

**Authors:** Caroline Klint Johannesen, Amanda Marie Egeskov-Cavling, Stine Lund, Zitta Barrella Harboe, Thea K. Fischer

**Affiliations:** a Department of Clinical Research, Nordsjaellands Hospital, Hilleroed, Denmark; b Department of Pediatrics, Nordsjaellands Hospital, Hilleroed, Denmark; c Department of Clinical Medicine, University of Copenhagen, Copenhagen, Denmark; d Department of Infectious and Pulmonary Diseases, Nordsjaellands Hospital, Hilleroed, Denmark; e PandemiX, Research Centre of Excellence, Grant Number DNRF170, Roskilde University, Roskilde, Denmark; f Department of Public Health, University of Copenhagen, Denmark

Respiratory syncytial virus (RSV) remains a leading cause of hospitalisation among infants worldwide, with the highest burden consistently observed during the first months of life. In temperate settings such as Denmark, RSV epidemics follow a highly regular seasonal pattern, a stability that re-emerged after the disruption caused by the COVID-19 pandemic.^1^ During the 2025-26 season RSV surveillance data from Denmark show a clear deviation from the expected epidemic curve (Fig. 1).

**Fig. 1:**
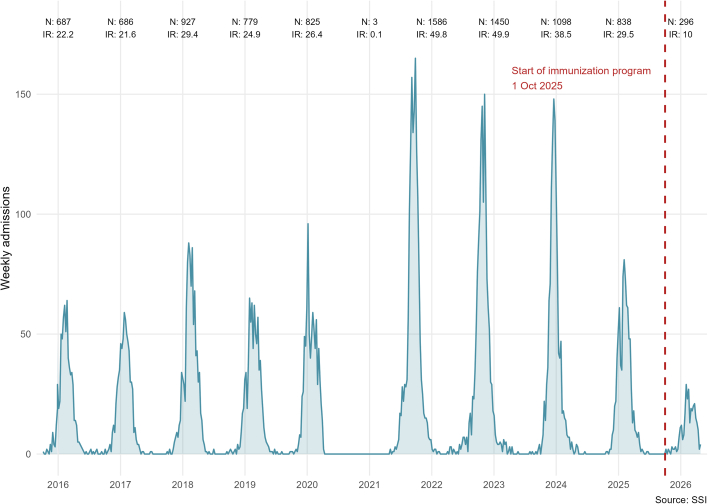
**RSV-associated hospitalizations in children 0–5 months, Denmark October 2015–April 2026.** Weekly admissions (light blue curve). Anotated with total admissions per season (N) and seasonal incidence rates (IR). Red line show introduction of immunization program (maternal vaccination and monoclonal antibodies). An RSV-associated hospitalisation is defined as a hospital admission lasting more than 12 h with a laboratory-confirmed RSV diagnosis recorded up to four days before or during admission. Incidence rates are based on the seasonal mean weekly age-specific population. Original figure. Data source: SSI. The red line indicates the introduction of the RSV immunisation programme (maternal vaccination and monoclonal antibodies).

In Denmark, RSV testing and RSV-associated hospital admissions are monitored through routine microbiological and hospital surveillance through discharge databases, and reported publicly by Statens Serum Institute. In surveillance, an RSV-associated hospitalisation is defined as a hospital admission lasting more than 12 h, with laboratory-confirmed RSV recorded within 4 days before or during admission.^1^ The surveillance has covered 11 seasons since 2015–16. Each season covers 52 weeks, starting in week 21 and ending in week 20.

Between the 2022–23 and 2024-25 Danish RSV seasons, the average incidence rate was 39·3 RSV-associated hospitalisations per 1000 infants aged 0–5 months (average n = 1128 per season). Though changes in RSV epidemiology were observed following the COVID-19 pandemic, the incidence rates of hospital admissions with RSV in young children have consistently been high in Denmark.^2^^,^^3^

On October 1, 2025, Denmark introduced a nationwide RSV prevention programme combining maternal vaccination with long-acting RSV monoclonal antibody prophylaxis (mAbs) for non-vaccinated high-risk and premature infants. Pregnant women gestational week 32 between October 2025 and February 2026 were offered vaccination, achieving an uptake of 72%.^4^

By April 29, 2026, 296 RSV-associated hospitalisations had been recorded among infants aged 0–5 months. An incidence rate of 9·9 admissions per 1000 children in the age group. Compared to the average observed across the three preceding seasons, the incidence rate ratio is 0·25 (95%CI: 0·22; 0·29). Weekly admission data show that this reduction occurred early and persisted throughout the winter.^1^

These ecological observations don't imply causality. Other explanations, like changes in healthcare behavior, testing, or viral interference, should be considered.

Similar early and sustained reductions have been reported following the introduction of RSV prevention programs in other European settings, including Spain, where universal use of mAbs has been associated with reductions in RSV-related hospitalizations of up to 80–85%, as well as in multi-country analyses from Belgium, Portugal and Spain showing around 79% effectiveness against RSV hospitalization. In England, maternal RSV vaccination has likewise been associated with reductions in infant RSV hospitalization exceeding 80%, supporting the plausibility of a program-related effect.^5^, ^6^, ^7^

However, the magnitude, age specificity, and timing of the reduction coincide closely with the introduction of systematic RSV prevention. RSV testing volumes remained substantial, while positivity rates declined markedly.

The age-specific decline occurred predominantly in infants targeted by the prevention program, argues against purely behavioral or testing-related explanations. Its effects appear to be population-wide. Comparable benefits have been demonstrated in clinical trials.^8^

If sustained, these findings suggest that large-scale maternal RSV immunization, combined with targeted mAbs use, can substantially reduce severe RSV disease in infants within a single season. Continued surveillance will be essential to assess durability and indirect effects in older children.

## Contributors

TKF, AMEC, and CKJ conceptualized the study, CKJ wrote the first version, and AMEC and TKF revised all versions. All authors have reviewed and approved the final version.

## Declaration of interests

CKJ had no conflicts of interest to declare. AMEC declare support from the Independent Research Fund Denmark outside of this work. SL had no conflicts of interest to declare. ZBH received research grants from Greater Copenhagen Health Science Partners (CAG VAX - Clinical Academic Group Translational Vaccine Research in High-Risk Adults), Independent Research Fund Denmark (Inge Lehmanns grant number 3162-00031B), Helen Rudes Foundation, the Danish Cancer Society (Grant number KBVU-MS R327-A19137), Novo Nordisk Foundation (grant nr. NNF24SA0090556), and the Danish National Research Foundation (grant number DNRF170), all out of this work. ZBH is an ID consultant at Statens Serum Institut, pneumococcal laboratory. ZBH functions as Chair, Vaccine Study Group (EVASG), European Society of Microbiology and Infectious Diseases (ESCMID) and is a member of the Danish Vaccination Council (NITAG). TKF declared consultant fees from a consultant company, grants or contracts from Pfizer, payment for lectures, support for meetings and/or travel from Pfizer and GSK. All payments from GSK and Pfizer were made to a consultant company.

## References

[1] Statens Serum Institut (2026). https://experience.arcgis.com/experience/220fef27d07d438889d651cc2e00076c/page/RS-virus.

[2] Johannesen C.K., van Wijhe M., Tong S. (2022). Age-specific estimates of respiratory syncytial virus-associated hospitalizations in 6 European countries: a time series analysis. J Infect Dis.

[3] Nygaard U., Holm M., Rabie H., Rytter M. (2024). The pattern of childhood infections during and after the COVID-19 pandemic. Lancet Child Adolesc Health.

[4] Statens Serum Institut (2026). https://www.ssi.dk/vaccinationer/tilslutning-til-bornevaccinationsprogrammet.

[5] Razzini J.L., Giné-Vázquez I., Jin J. (2026). Impact of universal nirsevimab prophylaxis in infants on hospital and primary care outcomes across two respiratory syncytial virus seasons in Galicia, Spain (NIRSE-GAL): a population-based prospective observational study. Lancet Infect Dis.

[6] Savulescu O., Ganser I., Nicolay N. (2025). Effectiveness of nirsevimab against RSV hospitalisation among children: a multicentre case–control study in Belgium, Portugal and Spain, 2024/25 season. Euro Surveill.

[7] UK Health Security Agency (2026). https://www.gov.uk/government/news/rsv-maternal-vaccine-cuts-baby-hospital-admissions-by-up-to-85.

[8] Lopes J.R., Martins Esteves I., Dias S.O. (2026). The impact of maternal respiratory syncytial virus vaccination on infant and perinatal outcomes: a systematic review and meta-analysis of randomized controlled trials. J Perinatol.

